# Brain Deletion of Insulin Receptor Substrate 2 Disrupts Hippocampal Synaptic Plasticity and Metaplasticity

**DOI:** 10.1371/journal.pone.0031124

**Published:** 2012-02-27

**Authors:** Derek A. Costello, Marc Claret, Hind Al-Qassab, Florian Plattner, Elaine E. Irvine, Agharul I. Choudhury, K. Peter Giese, Dominic J. Withers, Paola Pedarzani

**Affiliations:** 1 Research Department of Neuroscience, Physiology and Pharmacology, University College London, London, United Kingdom; 2 Department of Medicine, University College London, London, United Kingdom; 3 Wolfson Institute of Biomedical Research, University College London, London, United Kingdom; 4 Metabolic Signalling Group, MRC Clinical Sciences Centre, Imperial College London, London, United Kingdom; Centre national de la recherche scientifique, University of Bordeaux, France

## Abstract

**Objective:**

Diabetes mellitus is associated with cognitive deficits and an increased risk of dementia, particularly in the elderly. These deficits and the corresponding neurophysiological structural and functional alterations are linked to both metabolic and vascular changes, related to chronic hyperglycaemia, but probably also defects in insulin action in the brain. To elucidate the specific role of brain insulin signalling in neuronal functions that are relevant for cognitive processes we have investigated the behaviour of neurons and synaptic plasticity in the hippocampus of mice lacking the insulin receptor substrate protein 2 (IRS-2).

**Research Design and Methods:**

To study neuronal function and synaptic plasticity in the absence of confounding factors such as hyperglycaemia, we used a mouse model with a central nervous system- (CNS)-restricted deletion of IRS-2 (*NesCreIrs2KO*).

**Results:**

We report a deficit in NMDA receptor-dependent synaptic plasticity in the hippocampus of *NesCreIrs2KO* mice, with a concomitant loss of metaplasticity, the modulation of synaptic plasticity by the previous activity of a synapse. These plasticity changes are associated with reduced basal phosphorylation of the NMDA receptor subunit NR1 and of downstream targets of the PI3K pathway, the protein kinases Akt and GSK-3β.

**Conclusions:**

These findings reveal molecular and cellular mechanisms that might underlie cognitive deficits linked to specific defects of neuronal insulin signalling.

## Introduction

Substantial epidemiological evidence supports an association between diabetes mellitus and cognitive impairment [1]–[3]. Animal models of diabetes exhibit impaired learning and memory [4]–[8], effectively prevented by administration of insulin [4], [6]. Insulin, its related peptide, insulin-like growth factor-1 (IGF-1), and their receptors (IRs and IGF-1Rs) show abundant expression throughout the CNS. Especially high levels can be found in brain regions that are involved in higher cognitive functions, such as the hippocampus [9], [10]. However, diabetic rodent models and human patients are susceptible to suffer complex effects of systemic hyperglycaemia and glucose intolerance, such as vascular disorders, hypertension and heart disease, which can independently exacerbate cognitive impairment [1]. This makes it difficult to dissect the potential role of brain insulin signalling in cognition and its cellular and molecular mechanisms.

IR/IGF-1R are tyrosine kinases that activate downstream targets by phosphorylating insulin receptor substrate (IRS) proteins [11]–[14]. IRS-1 and IRS-2 are widely expressed in the brain [15]–[19]. Phosphorylation of IRS proteins leads to activation of the phosphatidylinositol-3 kinase (PI3K) and mitogen-activated protein kinase (MAPK/ERK) pathways [20]–[23]. Interestingly, these pathways are also involved in the induction/expression of hippocampal synaptic plasticity changes, such as long-term potentiation (LTP) [24]–[29], which is compromised in experimental models of diabetes [5], [6], [8], [30]–[34].

Although the contribution of specific IRS subtypes to neuronal synaptic function that is relevant for cognition has not been firmly established, previous work indicate a predominant role of IRS-2 in the control of brain anatomy and metabolic pathways that are important for synaptic plasticity and cognitive processes under normal and pathological conditions ([35]–[38]; but see [39]). In the present study, we have therefore questioned the role of neuronal IRS-2 in hippocampal synaptic function and plasticity by using a mouse model with a central nervous system- (CNS)-restricted deletion of IRS-2 (*NesCreIrs2KO*). In *NesCreIrs2KO* mice, IRS-2 is absent in CNS progenitor derived cells (neurons), while its expression is normal in other tissues [18], [40]. While mice globally lacking IRS-2 develop diabetes due to insulin resistance and pancreatic β cell dysfunction [41], *NesCreIrs2KO* mice do not suffer from overt and progressive diabetes due to preservation of pancreatic β-cell mass and insulin concentration [18], [40]. *NesCreIrs2KO* mice therefore permit the investigation of the role of IRS-2 signalling in neurons in the absence of some confounding factors such as systemic hyperglycemia.

## Results

### Deficiency in IRS-2 does not affect intrinsic excitability of hippocampal CA1 neurons

**Figure 1:**
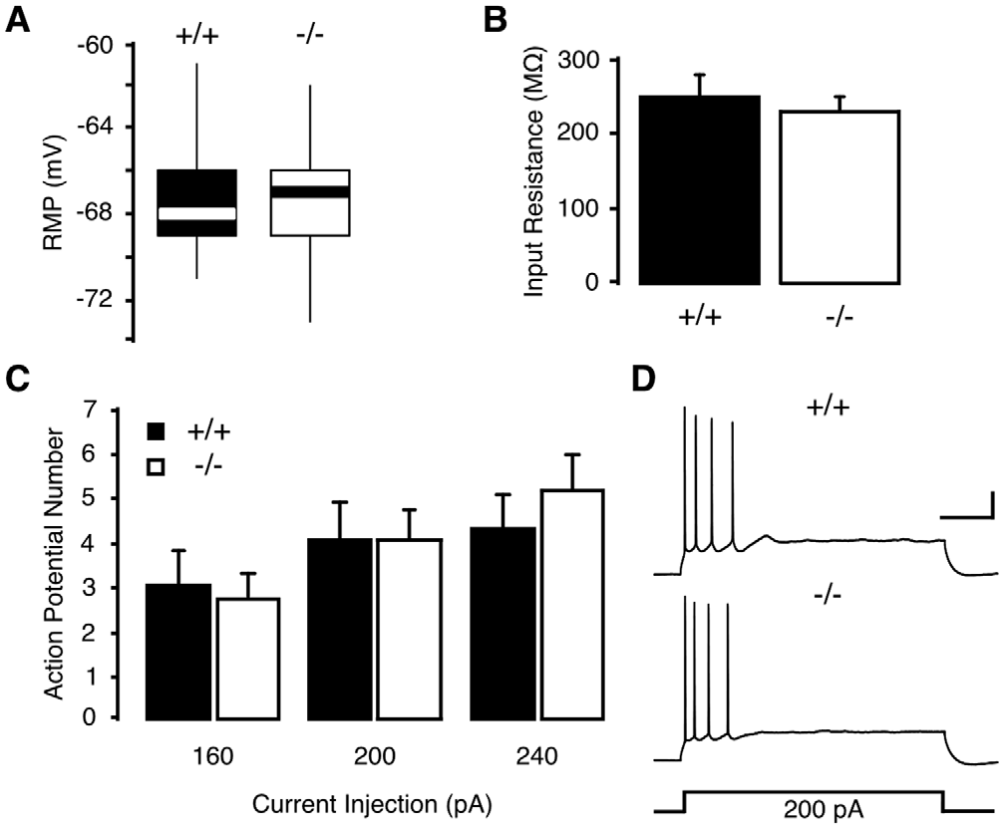
Intrinsic excitability of hippocampal neurons is not altered in adult *NesCreIrs2KO* mice. **A–B**: Neither resting membrane potential (RMP; **A**) or membrane resistance (Input Resistance; **B**) showed significant differences between CA1 pyramidal neurons of *NesCreIrs2KO* mice (−/−; −67±6 mV; 229±17 MΩ, N = 11, n = 20) and littermate controls (+/+; −67±5 mV; 248±27 MΩ, N = 14, n = 22). **C**: A similar number of action potentials were generated in response to 1 s-long current injections to CA1 neurons of control (+/+; 160 pA: 3.1±0.7; 200 pA: 4.1±0.8; 240 pA: 4.4±0.7) and *NesCreIrs2KO* (−/−; 160 pA: 2.8±0.5; 200 pA: 4.1±0.6; 240 pA: 5.2±0.8) mice. **D**: Sample traces taken from representative experiments in CA1 neurons of control (+/+; RMP: −58 mV) and *NesCreIrs2KO* (−/−; RMP: −60 mV) mice, illustrate the response to a 1 s-long injection of 200 pA. Scale bars: 20 mV, 200 ms.

Insulin/IGF-1 modulate membrane excitability and firing of hippocampal neurons by affecting various potassium and calcium conductances [42]–[46]. We therefore investigated whether neuronal IRS-2 regulates intrinsic membrane properties and firing patterns of hippocampal neurons. *NesCreIrs2KO* mice were generated and shown to lack IRS-2 expression in the brain [40]. No alteration in the hippocampal expression of IRS-1 was detected by RT-PCR in these animals (IRS-1 in *NesCreIrs2KO* mice: 99.3±14.2% of control; n = 5). Whole-cell recordings revealed no significant differences in the resting membrane potential (**Fig. 1A**) and membrane resistance (**Fig. 1B**) of CA1 pyramidal neurons from *NesCreIrs2KO* mice and littermate controls. Furthermore, there were no differences in neuronal firing in response to somatic current injections (**Fig. 1C**), instantaneous firing frequency (data not shown) and spike frequency adaptation (**Fig. 1D**). A brain-specific deficit in IRS-2 has therefore no significant impact upon the passive or active membrane properties of CA1 pyramidal neurons.

### NMDA receptor-mediated short-term plasticity (STP) is impaired in adult *NesCreIrs2KO* mice

**Figure 2:**
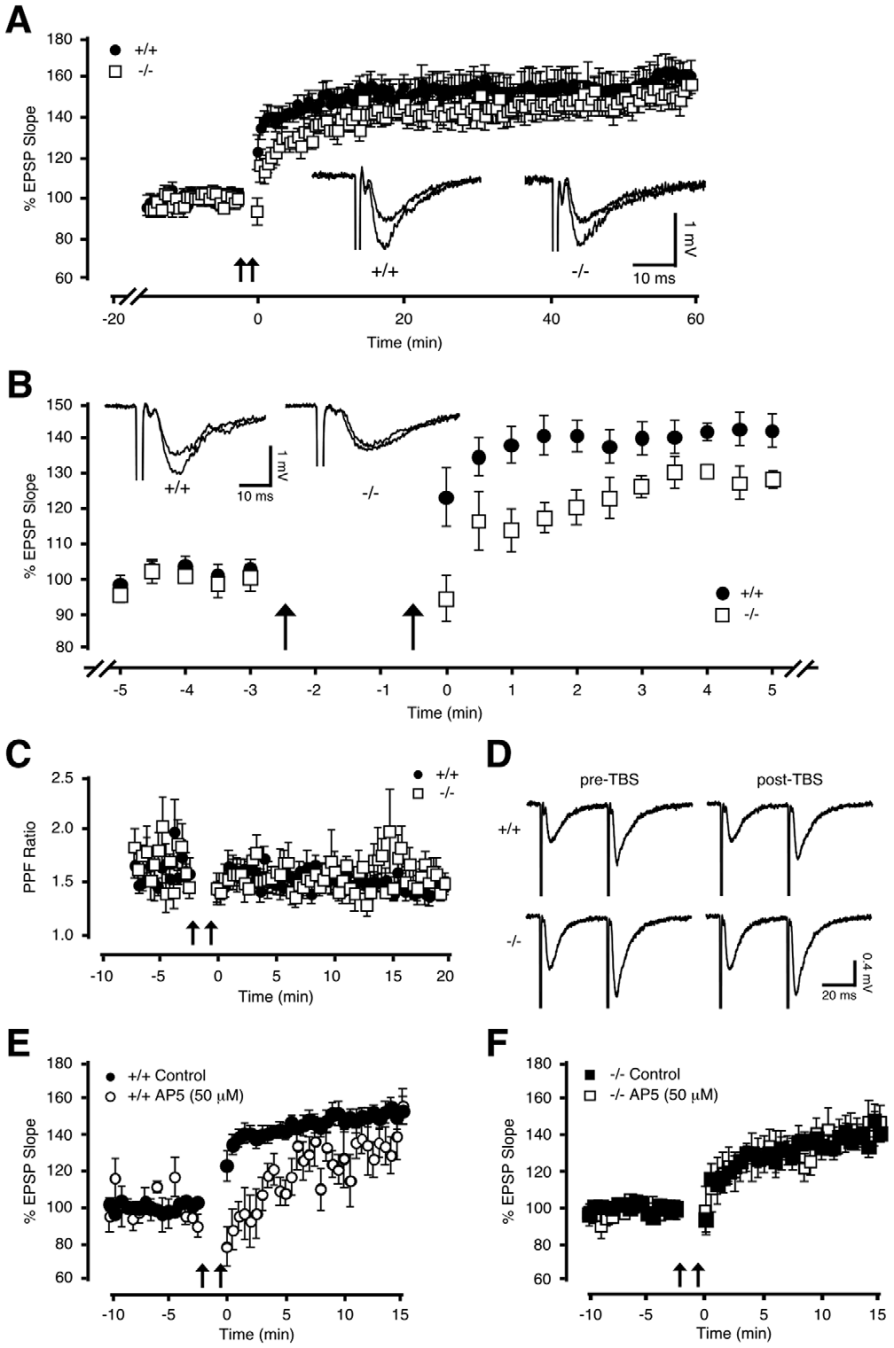
NMDA receptor-mediated short-term plasticity (STP) is impaired in adult *NesCreIrs2KO* mice. **A**: High-intensity theta burst stimulation (H-TBS) was applied to Schaffer collateral-commissural fibres following at least 15 minutes of stable baseline recording of synaptic activity in slices from 5–10 month old mice that had previously undergone behavioural training. In the absence of GABA receptor inhibitors, H-TBS induced robust long-term potentiation (LTP) of similar magnitude in both control (+/+; 160±7%, N = 7, n = 10) and *NesCreIrs2KO* mice (−/−; 152±6%, N = 5, n = 7), when measured 60 min following induction. Recordings made following H-TBS commence at time 0. **B**: Graph illustrates the same data as in **A**, but on an expanded time scale. The STP measured 2 min following H-TBS was significantly reduced in adult *NesCreIrs2KO* mice (−/−; average EPSP slope change: 110±5%, N = 5, n = 7) compared with that recorded from littermate controls (+/+; average EPSP slope change: 134±6%, N = 7, n = 10, p<0.01) during the same post-stimulus period. Insets illustrate typical EPSP traces (average of 4 consecutive sweeps) recorded immediately prior to, and either 60 min (**A**) or 2 min (**B**) following H-TBS in either control (+/+) or *NesCreIrs2KO* (−/−) mice. **C**: No significant change in paired-pulse facilitation (PPF) was observed following H-TBS in either control (pre H-TBS: 1.7±0.1; post H-TBS: 1.6±0.1; N = 5, n = 6) or *NesCreIrs2KO* mice (pre H-TBS: 1.6±0.2; post H-TBS: 1.5±0.1; N = 4, n = 5). Similarly, during the 2 min immediately following H-TBS, there was no significant difference in PPF between genotypes (+/+: 1.6±0.1; −/−: 1.5±0.1). **D**: Representative PPF traces (average of 4 consecutive sweeps) taken from single experiments carried out on a control (+/+) and *NesCreIrs2KO* (−/−) slice. EPSP traces are averages of 2 min recording immediately prior to, and following H-TBS. **E**: In the presence of the NMDA receptor antagonist DL-AP5 (50 µM), STP, measured 2 min following H-TBS, was significantly impaired in control mice (+/+ AP5; average EPSP slope change: 89±10%, N = 3, n = 5) compared with values obtained in the absence of DL-AP5 (+/+ control; average EPSP slope change: 134±6%, N = 7, n = 10, p<0.01). **F**: DL-AP5 had no significant effect on STP in *NesCreIrs2KO* mice (−/−) (−/− AP5; average EPSP slope change: 111±12%, N = 3, n = 5), compared with STP values obtained in the absence of DL-AP5 (−/− control; average EPSP slope change: 110±4%, N = 5, n = 6). Arrows indicate application of H-TBS.

Although insulin/IGF-1 have been shown to modulate glutamate receptor expression and activity [47]–[51] and synaptic plasticity [52]–[55], the downstream signalling pathways involved are not well understood. We specifically asked whether the IRS-2 deletion affected hippocampal synaptic transmission. Stimulation of the Schaffer collateral-commissural fibres (0.033 Hz) identified no difference in basal synaptic transmission at CA1 synapses of *NesCreIrs2KO* and control mice (data not shown). Long-term potentiation (LTP) is an activity-dependent model of synaptic plasticity, which is widely considered as a cellular correlate of learning and memory [56], [57] and is altered under conditions of deficient or defective insulin signalling [5], [6], [8], [30]–[34]. We assessed whether neuronal IRS-2 plays a specific role in LTP, elicited in CA1 synapses of adult mice (5–10 months old) by high-intensity theta burst stimulation (H-TBS, see Methods) of Schaffer collateral-commissural fibres. The H-TBS stimulation protocol reliably induced LTP in all slices from adult mice. The resulting LTP persisted for at least 60 minutes and was prevented by the application of the NMDA receptor antagonist DL-AP5 (100 µM) and Ca^2+^ channel blocker nimodipine (30 µM; n = 4; data not shown) in control mice. The LTP induced by H-TBS was of similar magnitude in control and *NesCreIrs2KO* mice (**Fig. 2A**). However, the short-term potentiation (STP) of the EPSPs measured 2 minutes after H-TBS was significantly reduced in *NesCreIrs2KO* mice (**Fig. 2B**). The difference in STP was not due to presynaptic changes, since paired-pulse facilitation (PPF) was unchanged before and after H-TBS in either control or *NesCreIrs2KO* mice, suggesting that the probability of neurotransmitter release was not modified by the IRS-2 deficiency (**Fig. 2C and D**). To further confirm that the STP following H-TBS was of postsynaptic origin and dependent on the activation of NMDA receptors, we applied H-TBS in the presence of DL-AP5 (50 µM). DL-AP5 significantly reduced STP in control mice (**Fig. 2E**). In contrast, in *NesCreIrs2KO* mice DL-AP5 did not affect the amplitude and slope of the EPSPs measured 2 min following the H-TBS (**Fig. 2F**). The lack of effect of DL-AP5 is likely to be due to an impairment of NMDA receptor function underlying the H-TBS-induced STP in *NesCreIrs2KO* mice.

### LTP and glutamatergic transmission are altered in CA1 synapses of adult *NesCreIrs2KO* mice

**Figure 3:**
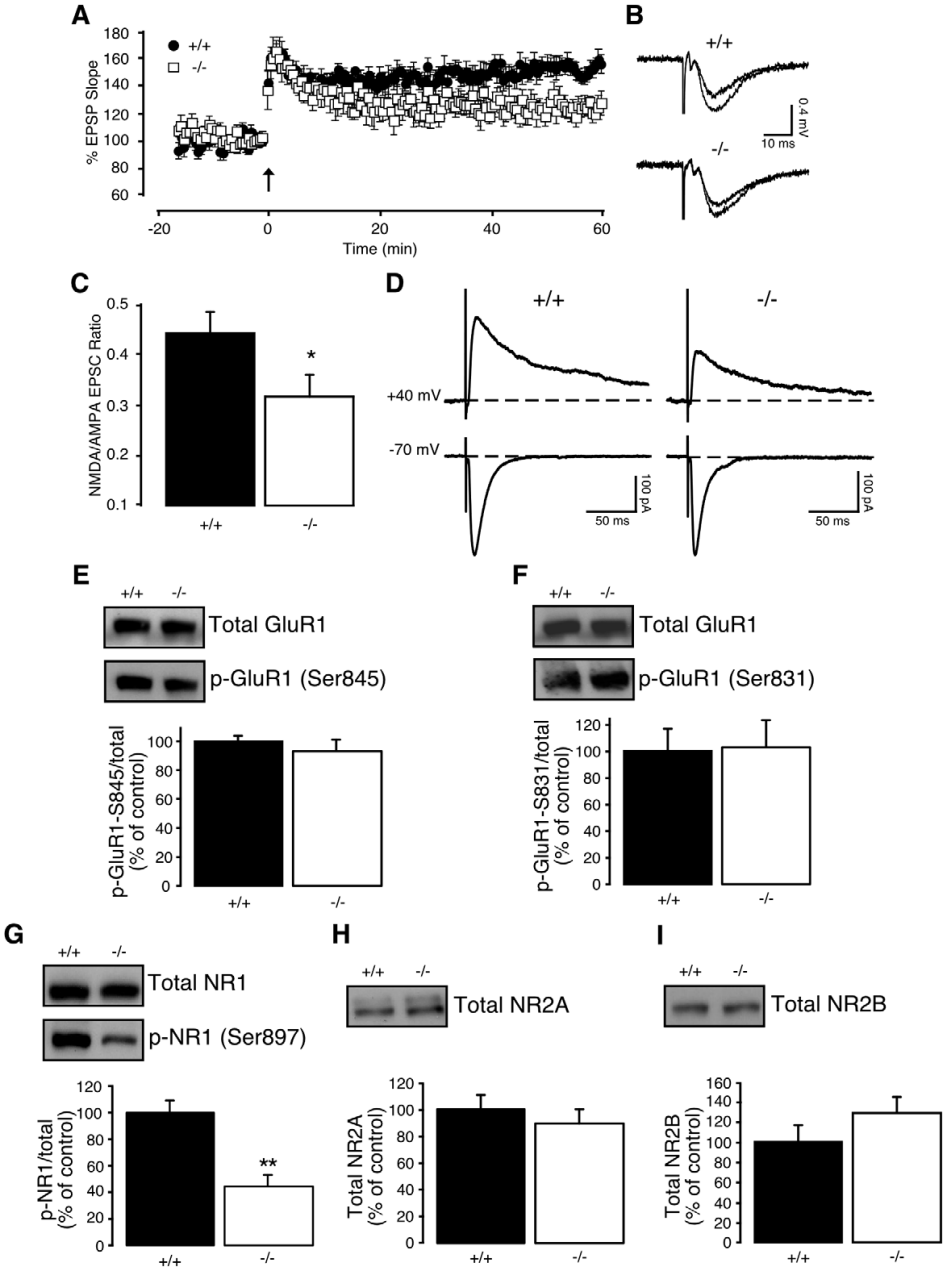
TBS-induced LTP and the NMDA- to AMPA-mediated EPSC ratio are reduced in adult *NesCreIrs2KO* mice. **A**: In the presence of the GABA_A_ receptor antagonist SR95531 (6 µM), LTP was reliably induced in control mice (+/+; average EPSP slope change 60 min after induction: 151±6%, N = 4, n = 6) in response to a single theta-burst protocol (TBS). The level of LTP produced under the same conditions, 60 min following TBS, was significantly reduced in *NesCreIrs2KO* mice (−/−; average EPSP slope change 60 min after induction: 123±6%, N = 4, n = 5, p<0.005). Arrow indicates application of TBS. **B**: Sample EPSP traces (average of 4 consecutive sweeps) from individual experiments, taken prior to, and 60 min following TBS in control (+/+) and *NesCreIrs2KO* (−/−) mice. **C**: In the presence of SR95531 (6 µM), the ratio of NMDA-EPSC amplitude (holding potential: +40 mV) to AMPA-EPSC amplitude (holding potential: −70 mV) was significantly lower in *NesCreIrs2KO* (−/−; 0.32±0.04, N = 6, n = 11) compared to littermate controls (+/+; 0.44±0.04, N = 5, n = 12, p<0.05). **D**: EPSC traces (average of 10 consecutive sweeps) from whole-cell patch clamp experiments in *NesCreIrs2KO* (−/−) and control mice (+/+) illustrating the NMDA-EPSC (measured at a holding potential of +40 mV) and corresponding AMPA-EPSC (measured at a holding potential of −70 mV). NMDA-EPSC amplitude was measured 50 ms following stimulation to minimize contamination from the AMPA-mediated component. **E–I**: Representative immunoblot images of hippocampal subfield CA1 lysates from control (+/+) and *NesCreIrs2KO* mice (−/−) probed with antibodies against the total protein or specific phosphorylation sites of the AMPA receptor subunit GluR1 (**E–F**) and the NMDA subunits NR1 (**G**), NR2A (**H**), and NR2B (**I**). Quantifications of immunoblots showing the protein level or relative proportion of phosphorylated protein over the total are shown in the bar diagrams. **E–F**: The total level of the AMPA receptor subunit GluR1 was similar in control (100±8%) and *NesCreIrs2KO* mice (77±9%, n = 10, p = 0.063; **upper panels**). **E**: The proportion of GluR1 subunit phosphorylated at Ser845 (p-GluR1-S845) relative to total GluR1 protein was similar in control (100±4%, n = 5) and *NesCreIrs2KO* mice (93±8%, n = 5, p = 0.46; **middle and lower panels**). **F**: The proportion of GluR1 subunit phosphorylated at Ser831 (p-GluR1-S831) relative to total GluR1 protein was similar in control (Ser831: 100±13%; n = 5) and *NesCreIrs2KO* mice (Ser831: 103±18%, n = 5; p = 0.73; **middle and lower panels**). **G**: There was no change in the total level of the NMDA receptor subunit NR1 (control mice: 100±11%; *NesCreIrs2KO* mice: 93±8%, n = 5, p = 0.46; **upper panel**). *NesCreIrs2KO* mice displayed a reduced phosphorylation of NR1 at Ser897 (p-NR1; 45±9%, n = 6) relative to their control littermates (100±9%, n = 6, p<0.001; **middle and lower panels**). **H–I**: There were no changes in the total levels of the NMDA receptor subunits NR2A (**H**; control mice: 100±11%; *NesCreIrs2KO* mice: 89±11%, n = 7, p = 0.49), and NR2B (**I**; control mice: 100±17%; *NesCreIrs2KO* mice: 129±17%, n = 5, p = 0.55). Data are expressed as mean ± SEM. **p<0.01 (unpaired t-test).

Next, we facilitated the induction of synaptic plasticity in adult mice by suppressing fast inhibitory synaptic transmission. Upon application of the GABA_A_ receptor antagonist SR95531 (6 µM), a single TBS was sufficient to produce a substantial LTP in CA1 synapses of control mice (**Fig. 3A and B** **, upper traces**). However, the level of LTP was significantly reduced in *NesCreIrs2KO* mice (**Fig. 3A and B** **, lower traces**). Therefore, in response to a weaker induction protocol delivered upon inhibition of GABA_A_ receptors, *NesCreIrs2KO* mice show an impaired level of LTP with respect to their control littermates.

NMDA receptor activation is essential for the induction of LTP at Schaffer collateral-CA1 synapses [58], [59], also in response to TBS [60]. Changes in NMDA receptor function and expression have been described in animal models of diabetes [61], [62] and as a direct response to application of insulin [47]–[51]. To test whether alterations in the NDMA-dependent synaptic transmission were responsible for the reduced LTP in mice lacking neuronal IRS-2, we analysed AMPA- and NMDA-mediated excitatory post-synaptic currents (EPSCs) evoked by stimulation of the Schaffer collateral-commissural fibres after blocking GABA_A_-mediated transmission and recorded at a holding potential of −70 mV and +40 mV, respectively. The ratio of NMDA- to AMPA-mediated EPSC amplitude was significantly lower in *NesCreIrs2KO* (**Fig. 3C and D** **, right**) compared to littermate controls (**Fig. 3C and D** **, left**). The lower ratio of NMDA- to AMPA-mediated EPSC amplitude in *NesCreIrs2KO* mice could be due to changes in either the expression or activity of AMPA and NMDA receptors. Spontaneous excitatory synaptic activity, corresponding to miniature and spontaneous action potential-driven events, was entirely dependent on AMPA receptor activation in our recordings, since it was fully suppressed by application of the AMPA receptor antagonist NBQX (5 µM). We analyzed the amplitude of all spontaneous events recorded at −70 mV (n = 12 cells from 5 wild-type and n = 11 cells from 6 *NesCreIrs2Ko* mice). The mean amplitude of spontaneous events in wild-type CA1 neurons was 13.5±0.3 pA (n = 438); in *NesCreIrs2Ko* cells, 13.4±0.3 pA (n = 561; p = 0.76). This indicates that basal synaptic AMPA receptor function is unlikely to be changed (i.e. increased) in *NesCreIrs2Ko* mice. Additionally, NBQX (5 µM) had a small effect on the EPSCs recorded at +40 mV, a potential at which the contribution by NMDA receptor activation is predominant. In 13 CA1 pyramidal neurons, NBQX caused a reduction in the current peak amplitude by 16.1±4.9%. The reduction caused by NBQX on wild-type cells (15.1±5.9%, n = 9) was not statistically different from that in *NesCreIrs2KO* cells (18.3±10.2%, n = 4; p = 0.78). Although indirect, this result suggests that NBQX had a similar impact on the EPSCs measured at +40 mV in wild-type and *NesCreIrs2KO* mice, supporting the notion that their AMPA-mediated component was similar. Finally, after assessing that the NMDA contribution to the amplitude of extracellularly recorded fEPSPs was negligible by evaluating the effects of DL-AP5 (50–100 µM) at different time points (change in fEPSP amplitude the presence of DL-AP5: peak, 8.4±4.6%, p = 0.11; 20 ms post-stimulus, 1.7±9.1%, p = 0.85; 30 ms post-stimulus, −12.4±6.4%, p = 0.09; n = 9), we compared input-output curves of fEPSPs obtained from control and *NesCreIrs2KO* mice. When plotted against stimulus intensity or indeed fibre volley amplitude, no strain-specific differences were observed (not shown). This finding further supports the idea that there is no difference in basal AMPA receptor function in *NesCreIrs2KO*. As a further, independent line of evidence highlighting potential differences in AMPA and NMDA receptors under basal conditions, we carried out a western-blot protein analysis in lysates from the hippocampal CA1 subfield of both control and *NesCreIrs2KO* mice. There was no significant alteration in the total amount of the AMPA receptor subunit GluR1 between genotypes (**Fig. 3E–F** **, upper panels**). Additionally, the proportion of phosphorylated GluR1 subunit (pGluR1) at Ser845 (**Fig. 3E**) and Ser831 (**Fig. 3F**) relative to total GluR1 protein was comparable in controls and *NesCreIrs2KO* mice. Similarly, there were no changes in the total levels of the NMDA receptor subunits NR1 (**Fig. 3G** **, upper panel**), NR2A (**Fig. 3H**) and NR2B (**Fig. 3I**). Conversely, *NesCreIrs2KO* mice displayed a significant reduction in the relative proportion of NR1 subunit phosphorylated at Ser897 (pNR1) relative to their control littermates (**Fig. 3G**). Since phosphorylation of NR1 at Ser897 is known to increase the activity of NMDA receptors [63]–[68], the reduced phosphorylation observed in *NesCreIrs2KO* mice may underlie the lower ratio of NMDA- to AMPA-mediated EPSC amplitude, and contribute to the attenuation of LTP in the absence of neuronal IRS-2.

### LTP and phosphorylation of Akt/protein kinase B and glycogen synthase kinase-3 (GSK-3) are impaired in CA1 of juvenile *NesCreIrs2KO* mice

**Figure 4:**
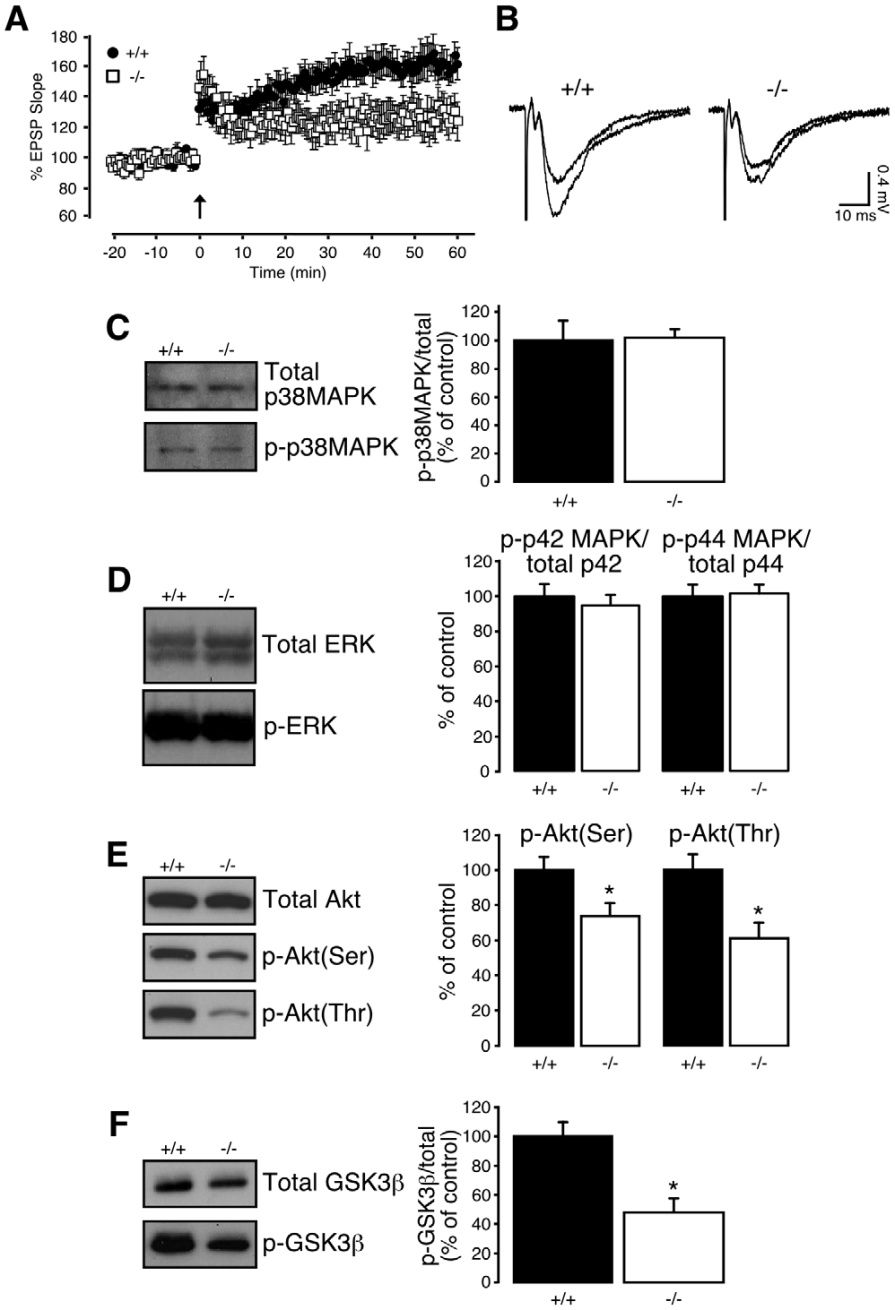
LTP and phosphorylation of Akt and GSK-3β are reduced in juvenile *NesCreIrs2KO* mice. **A**: LTP induced by a single TBS in slices with intact inhibitory synaptic transmission was significantly reduced in juvenile *NesCreIrs2KO* mice (−/−; average EPSP slope change: 128±11%, N = 5, n = 7) compared to values obtained from littermate controls (+/+; average EPSP slope change: 160±9%, N = 7, n = 7, p<0.05), 60 min following induction. **B**: Representative EPSP traces (average of 4 consecutive sweeps) from individual experiments taken immediately prior to TBS, and following 60 min of LTP in both control (+/+) and *NesCreIrs2KO* (−/−) mice. **C**: Immunoblot analysis from CA1 lysates showing that neither the total level of p38MAPK nor the amount of phosphorylated p38MAPK (Thr180/Tyr182) are altered by brain-deletion of IRS-2 in *NesCreIrs2KO* (−/−; 102±8%) mice compared with control littermates (+/+; 100±14%, n = 5–7; p = 0.91). The bar diagram on the right shows a summary of the data obtained from 5–7 mice per experimental group. **D**: Immunoblot analysis from CA1 specific lysates showing that neither the total level of p42/44 MAPK (ERK) nor the amount of ERK phosphorylated at Thr202 and Tyr204 are altered by brain-deletion of IRS-2 in *NesCreIrs2KO* mice. For p42 MAPK: +/+ 100±6.5%; −/− 94.8±5.5%; n = 5; p = 0.56. For p44 MAPK: +/+ 100±6.4%; −/− 101.5±4.7%; n = 5; p = 0.86. **E**: Immunoblot analysis from CA1 lysates showing reduced Akt/protein kinase B phosphorylation levels at Thr308 and Ser473 in *NesCreIrs2KO* mice (−/−; Ser473: 74±8.%; Thr308: 61±9%, n = 7) compared to controls (+/+; Ser473: 100±8%; Thr308: 100±9%, n = 7; p = 0.04 for Ser473, p = 0.01 for Thr308). The bar diagram on the right shows a summary of the data obtained from 7 mice per experimental group. **F**: Immunoblot analysis from CA1 lysates showing reduced phoshosphorylation of GSK-3β at Ser9 in *NesCreIrs2KO* mice (−/−; 48±10%, n = 5) compared to controls (+/+; 100±10%, n = 5; p = 0.007). The bar diagram on the right shows a summary of the data obtained from 5 mice per experimental group. Data are expressed as mean ± SEM. *p<0.05; **p<0.01 (unpaired t-test).

To assess whether IRS-2 affects NMDA-dependent synaptic plasticity also in the presence of intact inhibitory transmission, we performed recordings from younger, naive mice (3–6 weeks) in the absence of GABA_A_ receptor antagonists. A single TBS induced reliable LTP in control mice, but an attenuated one in juvenile *NesCreIrs2KO* mice (**Fig. 4A and B**). These results provide further indication that plasticity of CA1 synapses is impaired in *NesCreIrs2KO* mice in a way that is independent of age and experience.

The molecular mechanisms underlying NMDA-dependent LTP involve several protein kinases, including phosphatidylinositol 3-kinase (PI3K) and mitogen-activated protein kinases (MAPK/ERK) (for reviews see [57], [69], [70]). Activation of IRS proteins is known to regulate the activity of p42/44 MAPK (ERK), p38MAPK and PI3K [20], [21], [23], [71]–[73]. We therefore assessed whether the absence of IRS-2 in hippocampal neurons affects these signalling pathways. Under basal conditions, phosphorylation of p38MAPK at Thr180/Tyr182 was unaltered in CA1 lysates from *NesCreIrs2KO* compared to control mice (**Fig. 4C**). Similarly, Western blot analysis revealed that the activity of ERK, assessed by the phosphorylation levels at Thr202 and Tyr204, was similar in control and *NesCreIrs2KO* mice (**Fig. 4D**). Interestingly, however, phoshosphorylation of Akt/protein kinase B, a main downstream effector of PI3K, was substantially reduced at two distinct phosphorylation sites, Thr308 and Ser473, in *NesCreIrs2KO* mice (**Fig. 4E**). A prominent target for PI3K-Akt signalling is glycogen synthase kinase-3 (GSK-3), a regulator of hippocampal synaptic plasticity [74], [75]. We therefore investigated the phosphorylation state of the beta isoform of GSK-3 (GSK-3β) at Ser9, which has an inhibitory effect and is commonly used as a measure of GSK-3β activity [76], [77]. Consistent with a reduction in phospho-Akt, we found that phosphorylation of GSK-3β at Ser9 was significantly reduced in CA1 lysates of *NesCreIrs2KO* mice, suggesting an enhancement of GSK-3β activity (**Fig. 4F**). Our results show that the lack of neuronal IRS-2 is associated with a reduction in NMDA-dependent LTP at CA1 synapses. This plasticity deficit correlates with IRS-2-dependent alterations in the PI3K signalling pathway, leading to a reduced activity of Akt/protein kinase B and, consequently, to an increased GSK-3β activity in hippocampal neurons.

### Metaplasticity of CA1 synapses is inhibited by deletion of IRS-2

**Figure 5:**
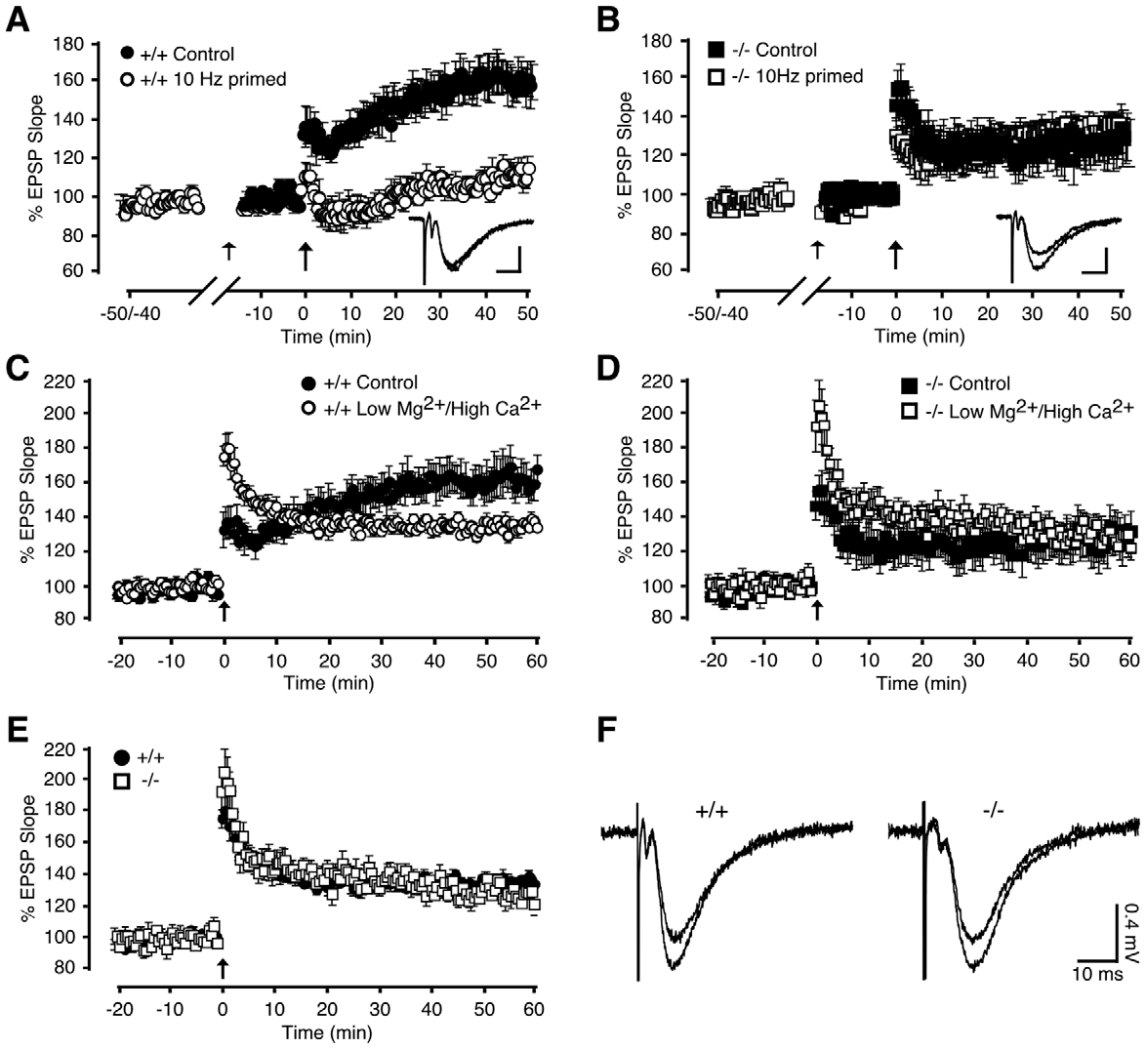
Metaplasticity cannot be induced in juvenile *NesCreIrs2KO* mice. **A**: A priming stimulus (10 Hz; small arrow) applied 20–30 min prior to TBS (large arrow) significantly inhibited subsequent LTP induction in juvenile control mice (+/+) for at least 50 min (average EPSP slope change: 10 Hz primed: 104±4%, N = 7, n = 7), when compared to un-primed LTP recorded 50 min following induction (average EPSP slope change: Control: 158±9%, N = 7, n = 7, p<0.0005). **B**: In juvenile *NesCreIrs2KO* mice (−/−), the same priming stimulus (10 Hz; small arrow) did not affect TBS-induced LTP (average EPSP slope change: 10 Hz primed; 135±5%, N = 7, n = 10), compared with the un-primed LTP measured 50 min post-TBS (average EPSP slope change: Control; 126±11%, N = 5, n = 7; p = 0.47). Insets in **A** and **B** show representative EPSP traces (average of 4 consecutive sweeps) taken prior to and 50 min following TBS, from individual experiments in control (**A**) and *NesCreIrs2KO* (**B**) slices to which 10 Hz priming stimuli had previously been applied. Scale bars: 0.4 mV; 10 ms. **C**: Enhancing NMDA receptor activity by lowering the concentration of extracellular Mg^2+^ to 1 mM and increasing the Ca^2+^ concentration to 3 mM, caused an attenuation of TBS-induced LTP in juvenile control mice (+/+) (average EPSP slope change: Low Mg^2+^/High Ca^2+^: 135±3%, N = 5, n = 6) compared with LTP recorded for 60 min under standard ionic conditions (average EPSP slope change: Control: 160±9%, N = 7, n = 7, p<0.05). **D**: The same ionic conditions to enhance NMDA receptor activity did not significantly alter the level of LTP obtained in *NesCreIrs2KO* mice (−/−) (average EPSP slope change: Low Mg^2+^/High Ca^2+^: 127±4, N = 4, n = 5) compared with that recorded in control conditions 60 min following induction (average EPSP slope change: Control: 128±11%, N = 5, n = 7). **E**: Metaplasticity induced in control mice (+/+) by altering extracellular divalent cation concentrations to enhance NMDA receptor activity yielded an LTP of similar magnitude to that observed in *NesCreIrs2KO* mice (−/−) under the same conditions. **F**: Representative EPSP traces (average of 4 consecutive sweeps) from individual experiments performed in low Mg^2+^/high Ca^2+^ conditions, taken prior to application of TBS and following 60 min of LTP in both control (+/+) and *NesCreIrs2KO* (−/−) mice.

At hippocampal synapses different activation patterns result in NMDA receptor signals that induce either LTP or long-term depression (LTD) of synaptic efficacy [56], [57], whose interplay can be critically regulated by GSK-3β [75]. Induction of NMDA-dependent LTP triggers the PI3K-Akt pathway, which in turn inhibits GSK-3β. In the inhibited state, GSK-3β can prevent the induction of LTD for up to one hour following LTP induction [75] in what can be regarded as a form of metaplasticity [78]. As GSK-3β activity is up-regulated in IRS-2-deficient neurons (**Fig. 4F**), it is conceivable that metaplasticity at CA1 synapses may be altered in *NesCreIrs2KO* mice. A priming stimulus (10 Hz) was applied to the synapses 20–30 minutes prior to TBS and did not itself produce a sustained alteration in synaptic efficacy, yet significantly inhibited subsequent LTP induction in juvenile control mice for at least 50 minutes (**Fig. 5A**). However, the same priming stimulus failed to alter the magnitude of LTP evoked in juvenile *NesCreIrs2KO* mice (**Fig. 5B**). This finding suggests that there is a deficit of metaplasticity in CA1 synapses of mice lacking neuronal IRS-2 in response to a conditioning stimulus capable of completely preventing LTP in control animals.

**Figure 6:**
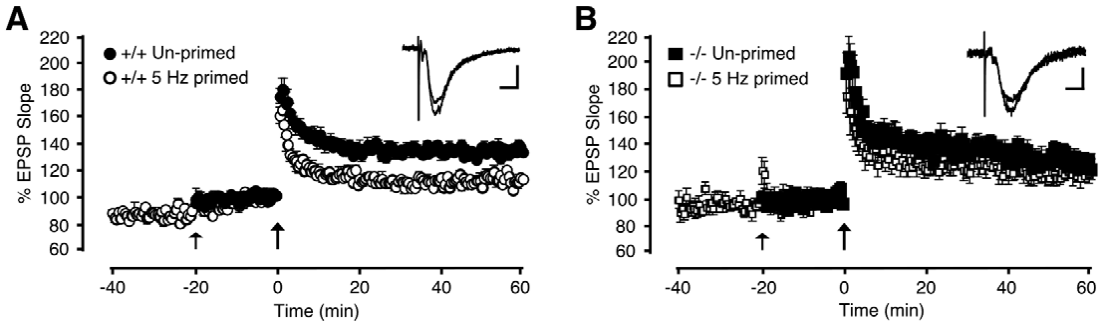
Metaplasticity cannot be induced in *NesCreIrs2KO* mice also under conditions that enhance NMDA receptor activity. **A**: A priming stimulus (5 Hz; small arrow) applied 20 min prior to TBS (large arrow) induced further metaplasticity in control mice (+/+) (5 Hz primed, 60 min post TBS; average EPSP slope change: 113±3%, N = 3, n = 5) compared with the un-primed values (Un-primed, 60 min post TBS; average EPSP slope change: 135±3%, N = 5, n = 6; p<0.00002), in the presence of 1 mM extracellular Mg^2+^ and 3 mM extracellular Ca^2+^ to enhance NMDA receptor activity. **B**: The same priming protocol did not significantly alter TBS-induced LTP in *NesCreIrs2KO* mice (−/−) (5 Hz primed, 60 min post TBS; average EPSP slope change: 120±6%, N = 3, n = 5) when compared to un-primed values recorded 60 min following induction (Un-primed, 60 min post TBS; average EPSP slope change: 127±4, N = 4, n = 5). Insets in **A** and **B** show representative EPSP traces (average of 4 consecutive sweeps) taken prior to and 60 min post-LTP induction. These traces are taken from individual experiments in control (**A**; +/+) and *NesCreIrs2KO* (**B**; −/−) slices to which 5 Hz priming stimuli had previously been applied in the presence of 1 mM extracellular Mg^2+^ and 3 mM extracellular Ca^2+^. Scale bars: **A**: 0.4 mV, 10 ms; **B**: 0.2 mV, 10 ms.

Common experimental procedures to induce metaplasticity involve pharmacological or synaptic activation of NMDA receptors [78]. The reduced contribution of NMDA receptor-mediated current to synaptically induced EPSCs in *NesCreIrs2KO* mice (**Fig. 3C and D**) might therefore play a role in the metaplasticity deficit observed in neurons lacking IRS-2 (**Fig. 5A and B**). We therefore asked whether pharmacological potentiation of NMDA receptor activity would induce metaplasticity in mice lacking neuronal IRS-2 by overcoming the partial loss of NMDA current in response to synaptic stimulation. To potentiate NMDA responses, the extracellular concentration of Mg^2+^ was lowered from 1.5 mM to 1 mM, and the extracellular concentration of Ca^2+^ raised from 2 mM to 3 mM. Under these conditions, LTP induced by a single TBS in CA1 synapses of juvenile control mice was substantially attenuated 60 minutes following induction (**Fig. 5C**). Under the same ionic conditions, however, no alteration in the level of LTP was observed in *NesCreIrs2KO* mice compared with standard ionic conditions (**Fig. 5D**). Interestingly, the magnitude of the LTP measured in control slices upon enhancement of NMDA receptor activity was similar to that recorded in *NesCreIrs2KO* mice (**Fig. 5E and F**). Stimulus-evoked metaplasticity could still be induced in control mice under conditions of increased NMDA receptor activity due to lower extracellular Mg^2+^ and higher concentration of Ca^2+^. Under these conditions, a short priming stimulus of lower frequency (5 Hz, applied 20 minutes prior to TBS) did not yield a sustained, significant alteration in synaptic transmission, but subsequently induced LTP was further reduced (**Fig. 6A**). This stimulus-induced metaplasticity was prevented by applying DL-AP5 (100 µM) during the priming period (n = 3; data not shown), confirming an involvement of NMDA receptors in its induction. Nonetheless, in *NesCreIrs2KO* synapses the same 5 Hz priming procedure failed to cause a reduction in subsequently induced LTP (**Fig. 6B**). Therefore, in mice lacking neuronal IRS-2 enhancing NMDA receptor activity did not change LTP compared to conditions where NMDA receptor activity was not enhanced, and did not induce any further metaplasticity. By contrast, enhancement of NMDA receptor activity was sufficient to induce metaplasticity in control animals, followed by a significant reduction in subsequently induced LTP. Moreover, *NesCreIrs2KO* mice lacked NMDA receptor-dependent, stimulus-evoked metaplasticity induced by low-frequency conditioning stimuli.

## Discussion

In this study, we have shown that brain deficiency of IRS-2 has a strong impact on NMDA receptor-dependent synaptic transmission, plasticity and metaplasticity in the CA1 region, which is associated with reduced basal phosphorylation of the NMDA receptor subunit NR1 and of downstream targets of the PI3K pathway, such as Akt/protein kinase B and GSK-3β.

Despite the reported effects of insulin/IGF-1-mediated signalling on the modulation of neuronal ion channels [42]–[46], [79], [80], deletion of IRS-2 does not appear to affect the intrinsic excitability of CA1 pyramidal neurons under basal conditions. Compensation by upregulation of IRS-1 is an unlikely explanation for this result, as levels of hippocampal IRS-1 RNA remain unchanged in *NesCreIrs2KO* mice. Our results therefore suggest that IRS-1 mediated signalling might be sufficient to maintain normal intrinsic excitability in CA1 pyramidal neurons.

The neuronal deficit in IRS-2 did however lead to a substantial impairment of NMDA receptor-mediated short-term (STP) and long-term potentiation (LTP) in Schaffer collateral-commissural synapses. Furthermore, we have identified a reduced contribution of NMDA receptors to glutamatergic transmission at CA1 synapses in *NesCreIrs2KO* mice, associated with a reduction in the basal level of phosphorylation of the NR1 subunit. It is well established that alterations in postsynaptic Ca^2+^ are necessary to evoke persistent changes in synaptic efficacy, such as LTP [81], [82]. In hippocampal CA1 synapses, NMDA receptor activation is a critical step in this process [58], [83]. The NR1 subunit is an integral component of all native NMDA receptors, and can be phosphorylated by protein kinases, such as PKC on Ser896 and PKA on Ser897, to potentiate receptor function [63]–[68], [84], [85]. The reduced phosphorylation of the NR1 subunit at Ser897 (**Fig. 3G**) is likely to lead to the decrease in activity of NMDA receptors observed in *NesCreIrs2KO* mice, and might be accountable, at least in part, for the impairment observed in synaptic plasticity. The deficit in hippocampal LTP correlates well to previous studies carried out on experimental models of diabetes [5], [6], [8], [30]–[34], in this case with the advantage that the restricted loss of IRS-2 in neurons eliminates hyperglycaemia as a confounding systemic complication associated with diabetes [86]–[88]. It is worthy to notice that a previous study has shown that IRS-2 deficient mice have enhanced hippocampal spatial reference memory, working memory and contextual- and cued-fear memory [89]. Our finding that basal excitatory synaptic transmission and LTP are intact in 5–10 months old, behaviourally trained *NesCreIrs2KO* mice (**Fig. 2A**) is compatible with a lack of deficit in hippocampal learning and memory in IRS-2 deficient mice. The plasticity deficits that we have characterized in this study were evident in younger, untrained animals (**Fig. 4**) or in older, trained ones upon suppression of GABAergic inhibition (**Fig. 3**). Considering the well documented facilitatory effect of insulin on GABA receptor surface expression and function [90]–[93], this leads us to speculate that a gradually developing, compensatory attenuation of inhibitory transmission might have contributed to the enhancement in hippocampal-dependent learning observed in *NesCreIrs2KO* mice [89]. This study establishes for the first time a direct role for IRS-2 in modulating NMDA receptor-dependent synaptic plasticity, via regulation of NR1 phosphorylation. However, facilitating NMDA receptor activity through manipulation of ionic conditions was not itself sufficient to bring the LTP in *NesCreIrs2KO* mice to the same level as observed in control animals under standard ionic conditions (compare Fig. 5C, closed symbols, and 5D, open symbols), revealing the involvement of downstream NMDA receptor-mediated molecular processes.

IRS-2 deficiency might indeed lead to deficits in NMDA-dependent hippocampal synaptic plasticity by causing multiple alterations of NMDA receptor post-translational modifications and function. While our study shows normal total levels of NR1, NR2A and NR2B subunits and a lower level of basal phosphorylation of NR1 at Ser897 in *NesCreIrs2Ko* mice, a study by Martin and colleagues [94], published while this paper was under revision, supports our findings on the total level of NR2A and NR2B subunits being normal in global IRS-2 KO mice. However, they found a reduced tyrosine phosphorylation of NR2B subunits following LTP induction and a reduced effect of the NR2B specific antagonist ifenprodil on NMDA-EPSCs in global IRS-2 KO mice [94]. The findings in our and in Martin's study [94] are largely complementary and provide convergent lines of evidence supporting NMDA receptor dysfunction as a consequence of IRS-2 deficiency and a potential cause for synaptic plasticity deficits in IRS-2 deficient mice.

The signal transduction pathways downstream of NMDA receptor activation, which underlie LTP, include the PI3K [26]–[28], [95], [96] and MAPK/ERK pathways [57], [69], [70]. Both the PI3K and MAPK/ERK pathways are further implicated in the insulin/IGF-1-mediated modulation of synaptic function in several neurons [54], [55], [97], [98], and are prominent targets of IRS proteins [20], [21], [23], [71]. Furthermore, in knockout mice expressing a brain-restricted insulin receptor deficiency (NIRKO) brain insulin resistance impairs insulin-mediated activation of either the PI3K/Akt/GSK-3β or MAPK/ERK pathways in cerebellar granule cells [23]. In *NesCreIrs2KO* mice the basal activity of p42/44 MAPK is not affected, while phosphorylation of the downstream target of PI3K, Akt/protein kinase B, is substantially reduced, providing a further potential mechanism for the impaired LTP observed in the absence of neuronal IRS-2. However, we cannot exclude that p42/44 MAPK phosphorylation might be reduced in response to LTP-inducing stimuli, thus also participating in the observed deficits in plasticity in IRS-2-deficient mice. This seems indeed to be the case in global IRS-2 KO mice, where activation of MAPK was not sustained 30 min after the induction of LTP [94].

The multifunctional enzyme GSK-3 has recently emerged as a regulator of hippocampal synaptic plasticity [74], [75]. The GSK-3β isoform, abundantly expressed in brain, has high constitutive activity due to tyrosine phosphorylation and is inactivated by further phosphorylation at Ser9. Activation of PI3K/Akt, such as that induced by insulin/IGF-1 during glycogen metabolism, can phosphorylate Ser9 and inhibit GSK-3β activity. Peineau and colleagues [75] demonstrated an essential role for GSK-3β activity in the induction of NMDA receptor-dependent LTD, while a mouse model over-expressing active GSK-3β exhibited attenuated LTP at CA1 synapses [74]. In keeping with these reports, our findings establish for the first time a link between IRS-2 and GSK-3β in neuronal function, and show that neuronal deficiency of IRS-2 leads to an enhanced activation of GSK-3β, which in turn is associated with impaired CA1 LTP.

Metaplasticity is the modulation in the extent or direction of synaptic plasticity by the previous activity of a synapse [78], [99], and it is thought to be important for cognitive processes and as a safeguard against excitotoxicity [78], [100]. In the hippocampus this corresponds to the facilitation or suppression of LTP or LTD due to prior activity-dependent priming of the same synapse induced through transient facilitation of NMDA receptor activity [101]. A striking finding is the inability to induce metaplasticity at CA1 synapses in *NesCreIrs2KO* mice, either through low-frequency stimuli or manipulation of ionic conditions to favour NMDA receptor activation. The molecular mechanisms underlying metaplasticity are still largely unknown, but a likely explanation for our finding is that, due to the tonic reduction in phospho-NR1, our conditioning stimuli might not have been sufficient to reach the threshold activation of NMDA receptors and rise in intracellular Ca^2+^ necessary to suppress subsequent LTP induction in *NesCreIrs2KO* mice. Finally, an intriguing aspect of GSK-3β signalling is its ability to modulate bi-directional plasticity. Indeed, the observation that LTP-inducing stimuli can inhibit GSK-3β activity and thereby prevent subsequent induction of LTD [75], suggests a role for this pathway in hippocampal metaplasticity. The profound reduction in metaplasticity and the concomitant dysregulation in GSK-3β basal phosphorylation in IRS-2 deficient mice shown in this study suggest a link between metaplasticity and GSK-3β function in the hippocampus. They further add IRS-2 signalling to the numerous pathways that have been shown to affect the dynamic range of neural networks involved in learning processes through the maintenance of a proper balance of LTP and LTD, and metaplasticity induction [78].

## Materials and Methods

### Animals

*Irs2lox* mice were intercrossed with *C57Bl6/J NesCre* mice (Jackson Laboratory) to generate compound heterozygous mice. Double heterozygous mice were crossed with *Irs2lox* mice to obtain wild-type, *Irs2lox/lox*, *Cre* and *CreIrs2lox/lox* mice. Mice lacking *Irs2* in nestin-expressing cells were designated *NesCreIrs2KO* [40]. Mice were handled in accordance to the Home Office Animal Procedures Act (1986) and University College London Animal Ethical Committee guidelines. All knockout and transgenic mice were studied with appropriate littermate controls.

### Hippocampal slice preparation

Acute hippocampal slices were prepared from control and *NesCreIrs2KO* mice of either sex, ranging in age from 3–6 weeks (**Fig. 4** **,** **5** **,** **6**) to 5–10 months (**Fig. 1** **,** **2** **,** **3**). Upon decapitation, brains were rapidly dissected and removed in ice-cold, oxygenated (95%O_2_/5%CO_2_) artificial cerebrospinal fluid (aCSF) containing (in mM): 125 NaCl, 1.25 KCl, 1 CaCl_2_, 1.5 MgCl_2_, 1.25 KH_2_PO_4_, 25 NaHCO_3_, and 10 D-glucose. Transverse, dorsal hippocampal slices (300 µm) were prepared using a VT1000S vibroslicer (Leica, Germany), and incubated in an interface chamber at room temperature for at least 1 hour prior to experimentation. Slices were transferred to a submersion recording chamber and superfused (2–3 ml/min) at room temperature with oxygenated aCSF containing (in mM): 125 NaCl, 1.25 KCl, 2 CaCl_2_, 1.5 MgCl_2_, 1.25 KH_2_PO_4_, 25 NaHCO_3_, and 10 D-glucose. A subset of experiments (**Fig. 5C–F** **and** **Fig. 6**) was performed with lower extracellular MgCl_2_ (1 mM) and higher CaCl_2_ (3 mM) concentrations. For experiments involving inhibition of GABA_A_ receptor-mediated transmission, a surgical incision was made between the CA3 and CA1 regions to minimise the propagation of epileptiform activity. Older animals (5–10 months; **Fig. 1** **,** **2** **,** **3**) had previously undergone behavioural training, at least 1 month prior to slice preparation.

### Whole-cell electrophysiological recordings

Gigaseal whole-cell recordings were obtained from somata of CA1 pyramidal neurons. Borosilicate glass patch electrodes (4.5–6.5 MΩ) were filled with an intracellular solution containing either (in mM): 135 potassium gluconate, 10 KCl, 10 HEPES, 2 Na_2_-ATP, 0.4 Na_3_-GTP, and 1 MgCl_2_, or (in mM): 135 cesium gluconate, 10 NaCl, 10 HEPES, 1 MgCl_2_, 2 Na_2_-ATP, 0.4 Na_3_-GTP, 3 EGTA; pH was adjusted to 7.2–7.3 with KOH (potassium gluconate solution) or NaOH (cesium gluconate solution); osmolarity 280–300 mOsm. Once in the whole-cell configuration, access resistance was regularly monitored and maintained at ≤25 MΩ. Only cells with an initial resting membrane potential between −55 and −75 mV were used, with no correction made for liquid junction potential. Membrane resistance was determined in response to a hyperpolarising step from −50 to −55 mV. Action potentials were evoked by 1 s-long current injections (40–400 pA). When recording whole-cell excitatory postsynaptic currents, series resistance was compensated by 50–75%. The Schaffer collateral-commissural pathway was stimulated (0.05 Hz) using an extracellular electrode (∼2 MΩ) filled with aCSF. Neurons were held in the voltage-clamp mode, at potentials of +40 mV or −70 mV. All recordings were carried out at room temperature (∼22–23°C).

### Extracellular electrophysiological recordings

The Schaffer collateral-commissural pathway was stimulated at 0.033 Hz (synaptic transmission) or 0.05 Hz (paired-pulse facilitation) and field excitatory postsynaptic potentials (EPSPs) recorded from the CA1 stratum radiatum using extracellular electrodes (described above). The stimulus intensity was adjusted to produce a response ∼50% of maximal EPSP amplitude as determined from input–output curves performed at the beginning of each experiment. A stable baseline of at least 10–20 min was recorded prior to pharmacological manipulation or application of plasticity-inducing stimuli. Paired-pulse facilitation was evoked by 2 consecutive pulses of 0.2 ms duration, at an interval of 50 ms. Two theta-burst stimulation protocols were used: the standard theta-burst stimulation (TBS) consisted of 6 trains (4 pulses at 100 Hz) repeated at 5 Hz; the high-intensity theta burst stimulation (H-TBS) corresponded to the TBS repeated twice with a 2 min interval. Metaplasticity was evoked by a 10 Hz (10 pulses) priming stimulus, applied once or twice at a 2 min interval, 20–30 min prior to TBS (**Fig. 5A,B**), or by 5 Hz trains (5 pulses repeated 20 times at 5 Hz intervals), 20 min prior to TBS (**Fig. 6**). All recordings were carried out at room temperature (∼22–23°C).

### Data acquisition and analysis

All data were acquired using an EPC10 amplifier and Patchmaster software (HEKA). Action potential trains were filtered at 2.5 kHz and sampled at 12.5 kHz. Synaptic potentials were filtered at 5 kHz and sampled at 20 kHz. Analysis was carried out offline using PulseFit (HEKA), Igor Pro 4.03A and Neuromatic (Wavemetrics Inc.; Dr. J. Rothman). Instantaneous firing frequency was determined by the reciprocal of the inter-spike interval between consecutive action potentials. Spike frequency adaptation was assessed as interval between the penultimate and last action potentials, divided by that of the first and second action potentials. Synaptic efficacy was determined as the measure of EPSP slope (10 to 80% of EPSP) or EPSC amplitude. LTP was assessed as percentage EPSP slope, normalised to the mean slope recorded during 5 minutes immediately prior to LTP induction. The magnitude of LTP was determined as the mean % EPSP slope recorded during the last 5 minutes of recording (either 50 min or 60 min post-TBS). PPF was assessed as the ratio of the slope of the 2^nd^ EPSP/1^st^ EPSP. AMPA receptor-EPSC amplitude was measured as the average peak of 10 consecutive EPSC traces, evoked at a holding potential of −70 mV. NMDA receptor-mediated EPSC amplitude was measured as the average of 10 consecutive EPSC traces at +40 mV 50 ms post-stimulus, to minimize contamination from AMPA receptor-mediated responses (see for example [102]–[104]). Statistical comparisons were made with two-tailed Student's t-tests (paired or unpaired as appropriate), using Excel (Microsoft) and InStat (GraphPad). “N” indicates the number of animals and “n” the number of experiments (extra- or intra-cellular recordings). In all cases, p<0.05 was considered significant. “*” indicates p<0.05.

### Protein lysate preparation and Western-blot analysis

Protein lysates were prepared from the microdissected hippocampal subfield CA1 at 4°C as previously described [105]. Protein concentrations were determined by BCA assay (Perbio) using bovine serum albumin (BSA) as standard. Equal amounts of protein were separated on SDS-polyacrylamide gel electrophoresis (PAGE) (pre-cast 5% or 10% polyacrylamide gels from Bio-Rad) followed by electrophoretic transfer to polyvinyl difluoride membranes (Amersham). Membranes were blocked with 5% non-fat dried milk in Tris-buffered saline at room temperature and incubated over-night at 4°C with primary antibodies. Horseradish peroxidase-conjugated secondary antibodies (Amersham) were used as appropriate and membranes developed with ECL Plus (Amersham). After detection of phosphoproteins, membranes were stripped using Restore Western Blot Stripping Buffer (Pierce) and reprobed with antibodies against non-phosphorylated proteins. Band intensities were quantified using the ImageJ software. Phosphoprotein levels were normalized to total protein levels and expressed as percent values relative to the control group. “n” indicates number of mice per experimental group. Statistical comparisons were made with two-tailed, unpaired Student's t-tests, using GraphPad Prism. “*” indicates p<0.05; “**” indicates p<0.01. The following primary antibodies were used: anti phospho Akt (Thr308 and Ser473, 1∶1000), anti Akt (1∶1000), anti phospho-GSK3β (Ser9, 1∶1000), anti GSK3β (1∶1000), anti phospho-p42/44 MAPK (Thr202/Tyr204), anti p42/44 MAPK (1∶1000), anti phospho-p38 MAPK (Thr180/Tyr182) and anti p38 MAPK (1∶1000) from Cell Signaling Technology; anti phospho NR1 (Ser897, 1∶3000), anti NR1 (1∶2000), anti NR2A (1∶2500) and anti GLUR1 (1∶10,000) from Upstate Biotech; phospho GLUR1 (Ser 831, Ser845, 1∶2000) and anti NR2B (1∶2500) from Chemicon.
